# Morphological Discrepancy of Various Basic Oxygen Furnace Steel Slags and Road Performance of Corresponding Asphalt Mixtures

**DOI:** 10.3390/ma12142322

**Published:** 2019-07-21

**Authors:** Yong Ye, Shaopeng Wu, Chao Li, Dezhi Kong, Benan Shu

**Affiliations:** State Key Laboratory of Silicate Materials for Architectures, Wuhan University of Technology, Wuhan 430070, China

**Keywords:** basic oxygen furnace steel slag, morphological discrepancy, road performance, mechanism research

## Abstract

Due to the difference of cooling and treatment processes (rolling method, hot braised method, layer pouring method), basic oxygen furnace (BOF) steel slag can be mainly classified as roller steel slag (RSS), hot braised steel slag (HBSS) and layer pouring steel slag (LPSS). Treatment difference directly results in the performance variations of different BOF steel slag and corresponding asphalt mixtures. The primary purpose of this research was to examine the effects of different cooling and treatment processes on the morphological discrepancy of different BOF steel slag. Also, the road performances of corresponding asphalt mixtures, and mechanism between steel slag performance and road performance were studied. The results show that LPSS owns the largest variability of angular index and texture index, and RSS has the most balanced morphological parameters. The structure of RSS asphalt mixture is advantageous for improving the ability of the asphalt mixture to resist the deformation and enhancing the stability of structure. Higher content of CaO and lower content of SiO_2_ make the acid-base reaction of RSS asphalt mixture most intense, which contribute to the best road performance of it.

## 1. Introduction

In recent years, rapid development of transportation infrastructure has aroused various obstacles [1], the lack of substantive natural aggregates which possess significant qualities is the most urgent one [2]. Substantive natural aggregates made asphalt mixtures inferior in moisture damage, crack resistance and rutting deformation in road construction [3,4]. The lack of them has made researchers to take other measures to alleviate the crisis, like the use of industrial waste [5].

Steel slag, one type of industrial wastes to replace superior natural aggregates, can be mainly classified as basic oxygen furnace (BOF) steel slag, electric arc furnace (EAF) steel slag, ladle refining (LF) steel slag, and casting residue according to the manufacturing types of steel production [2,6]. Among these, BOF steel slag has been widely utilized in producing numerous types of asphalt mixtures according to former researches [5,7,8]. Kambole reviewed common physical and mechanical characteristics of BOF slag and natural aggregates, and evaluated the influence of main aggregate performance on the properties of asphalt mixtures [9]. Results demonstrated that BOF steel slag owns very good technical and physical properties compared with natural stone aggregates, and proved its feasibility as a valuable resource for road pavements. In addition, mixtures of bitumen with BOF steel slag have yielded better resilient moduli, rutting resistance, bonding, moisture damage resistance and stripping resistance compared to mixes with natural aggregates [9,10,11]. López-Díaz used the BOF steel slag as coarse aggregate in asphalt concrete, and the results confirmed that it was feasible to use BOF steel slag to partially replace conventional aggregates in road paving [12]. Qazizadeh evaluated the effects of BOF steel slags on the fatigue behavior of asphalt mixes and found that the addition of slags in the mixtures considerably enhanced fatigue life of asphalt mixes [13]. Xue explored the feasibility of using BOF steel slag as aggregates in stone mastic asphalt (SMA) mixtures, results indicated that BOF steel slag improved the high-temperature stability and the low-temperature cracking resistance of SMA mixture when compared with basalt [14]. Shen studied the influence of porous asphalt mixture contained BOF steel slag on the mixture performance and sound absorption characteristic, results demonstrated that BOF steel slag enhanced the skid resistance, moisture susceptibility, rutting resistance, and sound absorption of porous asphalt mixtures [15].

Nevertheless, former researches neglected the influence of cooling and treatment techniques on the properties of BOF steel slag after being produced from furnace, and just placed different BOF steel slag in the same bracket indistinctly. Actually on the basis of different cooling and treatment techniques in China, the BOF steel slag can be mainly classified as roller steel slag (RSS), hot braised steel slag (HBSS) and layer pouring steel slag (LPSS) [16]. To obtain the RSS, firstly the liquid BOF steel slag is dumped into the rotating roller along the chute, steel balls are then added into the roller. By controlling the amount of water, finally steel slag can undergo heating, pulverizing, grinding, and cooling in the roller. The manufacture of HBSS is in accordance with the steps that liquid BOF steel slag is firstly dumped into the pit with the sprinkler and cover, and then smashed by mixing many saturated steams in the confined pit. Comparing to the former two treatments and cooling processes, the production of LPSS is much easier. The liquid BOF steel slag only needs to be pumped onto the slag bed (or inside the slag pit) firstly. Then, a proper amount of water is sprayed and LPSS is weathered in the natural environment.

Above treatment difference directly results in the performance variations of different BOF steel slag, such as the chemical composition, and morphology [17]. The chemical composition and morphology are directly related to the road performance of corresponding asphalt mixtures [18,19]. Therefore, in line with the properties aroused by different cooling and treatment techniques to select suitable application, this paper focused on the utilization of three BOF steel slags in asphalt mixtures, including RSS, HBSS, and LPSS, so that the BOF steel slag could have a broader utilization prospects in transportation infrastructure based on their difference. Firstly, both the surface texture of three BOF steel slags were investigated. Secondly, their chemical composition and morphological discrepancy were also included. Thirdly, three types of asphalt mixtures contained different steel slags were designed, and their road performances were tested. Finally, the mechanism between the BOF steel slag performance and road performance were also analyzed.

## 2. Materials and Methods

### 2.1. Raw Materials

**Figure 1:**
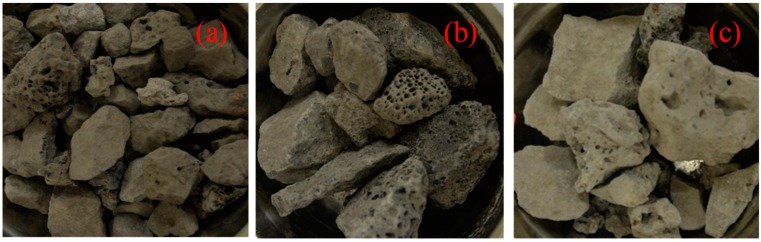
Appearance of different basic oxygen furnace (BOF) steel slag: (**a**) RSS; (**b**) LPSS; (**c**) HBSS.

The asphalt binder with penetration of 68.3 (0.1 mm at 25 °C), ductility of 151 cm (5 cm/min, 15 °C), and softening point of 47.5 °C, which supplied by Guochuang Co., Ltd., Wuhan, China, was used in this research [20]. Limestone aggregates with different particle size (0 mm–2.36 mm, 2.36 mm–4.75 mm, 4.75 mm–9.5 mm) and filler were supplied by Agoura Stone Processing Factory, Inner Mongolia and their properties were all met the standard specifications. Three types of basic oxygen furnace (BOF) steel slag, including roller steel slag (RSS), hot braised steel slag (HBSS), and layer pouring steel slag (LPSS), were supplied by Baotou Steel with particle size ranging from 9.5 mm to 19 mm, the basic properties of them were shown in Table 1. From the results of Los Angeles abrasion and crushing value, it can be seen that the mechanical properties of steel slag are better than natural aggregates. As these mechanical properties can reflect the hardness, wear resistance and anti-slip properties of aggregates closely related to the performance of asphalt mixtures [9]. Additionally, as shown in Figure 1, it can be seen that the three types of steel slag all have irregular shapes and porous structure. Among them, the surface texture of LPSS is the most abundant. This is because the aging process of LPSS occurs in natural conditions with no high temperature and high-pressure treatments. Therefore, the formation process of LPSS is uneven, slow and the pores are not fully filled with aged materials, which results in a topographical feature of the surface texture.

### 2.2. Asphalt Mixture Design

**Figure 2:**
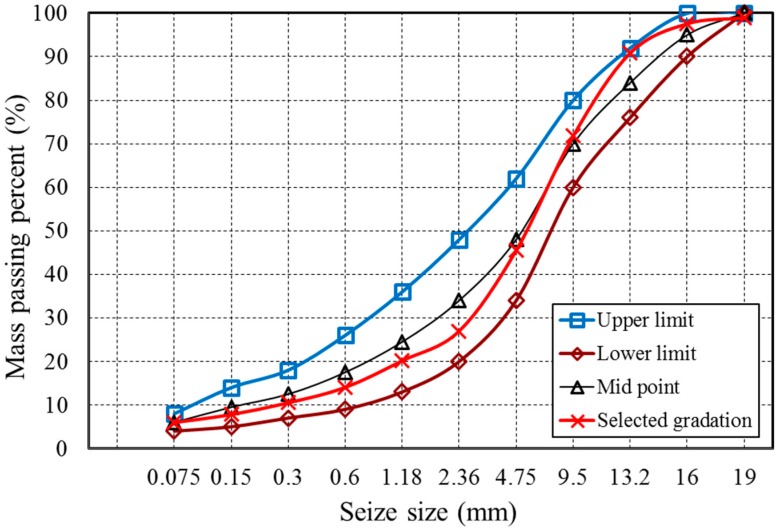
Gradation curve of AC-13 asphalt mixtures.

**Figure 3:**
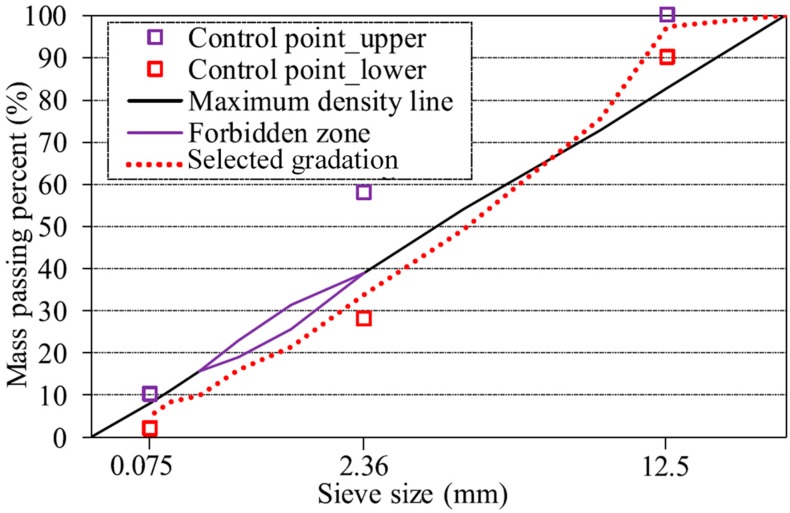
Gradation curve of Superpave-13 asphalt mixtures.

In this research, two types of asphalt mixtures were designed based on standard Marshall Method and Superpave method. Three types of AC-13 asphalt mixtures were mixed with same limestone aggregates, asphalt binder and different BOF steel slag, the gradation curve was shown in Figure 2. Additionally, three types of Superpave-13 asphalt mixtures were mixed the same materials as the AC-13 asphalt mixtures, and the gradation curve was shown in Figure 3.

### 2.3. Experimental Methods

#### 2.3.1. Characterization

The surface textures of different steel slags were detected by a JSM-5610LV Scan Electronic Microscope (SEM) manufactured by JEOL, Tokyo, Japan [25]. The resolution of SEM in the high-vacuum and low-vacuum mode was 3.0 nm and 4.0 nm separately; the magnification of 1000× was adopted in this research. Chemical composition changes of different steel slags were determined using an AXIOS X-ray fluorescence spectrometer (XRF) manufactured by PANalytical B.V., Amsterdam, The Netherlands [26].

#### 2.3.2. Morphological Discrepancy

AFA2 Aggregate Imaging System (AIMS) (PINE, New York, USA) was utilized to analyze the morphological properties of steel slag in this research, including angularity and texture. AIMS captures images of aggregates at different resolutions through a simple setup that consists of one camera and two different types of lighting schemes [27,28]. The image acquisition setup is configured to capture a typical image of 640 by 480 pixels at these resolutions in order to analyze the aggregates, and export the angularity index and texture index.

#### 2.3.3. Road Performance

In this paper, high temperature stabilities of AC-13 asphalt mixtures were performed by using the wheel tracking device, which was normally used for HMA testing and the dimension of tested slab specimen was 300 mm × 300 mm × 50 mm [29]. A solid rubber wheel with a wheel pressure of 0.7 MPa is used to walk on the specimen at 60 °C. When measuring the deformation period of the test piece, the dynamic stability, which means the number of times it needs to walk for every 1 mm augment, was obtained to evaluate the high temperature stabilities [30].

Based on ASTM D1075 and AASHTO T283, the moisture resistance abilities of three steel slag asphalt mixtures were evaluated by the water immersion Marshall test and the freeze-thaw split test. Both AC-13 asphalt mixtures and Superpave-13 asphalt mixtures were evaluated [31].

To evaluate the low temperature cracking resistance abilities of AC-13 asphalt mixtures, specimen was loaded under a universal material testing machine (IPC, Sydney, Australia) based on the single axes compression test at 0 °C, and the stress–strain curves were obtained automatically by the data acquisition system. The maximum compressive strain energy density was used to evaluate the low temperature cracking resistance abilities and calculated according to the Equation (1) [14].

(1) $$\frac{dW}{dV}=\int_{0}^{\varepsilon_{0}}\sigma_{ij}d\epsilon_{ij}$$

where *dW/dV* represents the compressive strain energy density function; *σ_ij_* represents the stress fraction; *ε_ij_* represents the strain fraction; and *ε*_0_ represents the strain at maximum compressive stress. 

## 3. Results and Discussions

### 3.1. Characterization

**Figure 4:**
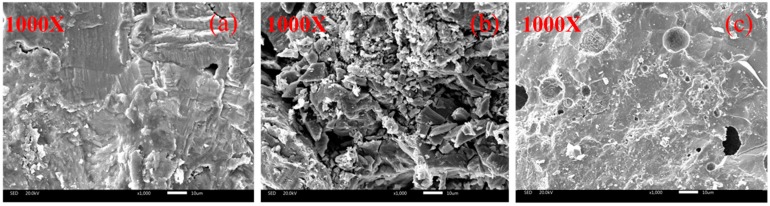
SEM results of different steel slags: (**a**) RSS; (**b**) LPSS; (**c**) HBSS.

The SEM results of different types of steel slags are shown in Figure 4, it can be indicated that all steel slags have rough surface and numerous pores. This morphological property contributes to improve the bonding performance between aggregate and asphalt binder, resulting in the enhancement of asphalt concrete structural stability. The shape characteristics of RSS and HBSS are similar with little pits and gullies on the surface, which are relatively smoother than the surface texture of LPSS. LPSS not only contains numerous pits, but also has a very uneven surface contained crystal materials with regular shapes. This is because aging process of LPSS is in the natural environment, in which the free calcium oxide slowly forms a calcium carbonate with a crystal structure under the action of water and carbon dioxide. The formed calcium carbonate crystal gradually fills the original pores of LPSS, and due to the uneven distribution of the free material on the surface of LPSS, large amounts of pores still exist on the surface of LPSS. In contrast, under the condition of high temperature and high pressure, RSS and HBSS have been fully aged, the resulting aged products are fully fused with original components of steel slag and formed into a whole, so the surface textures of RSS and HBSS are relatively smoother than LPSS.

**Figure 5:**
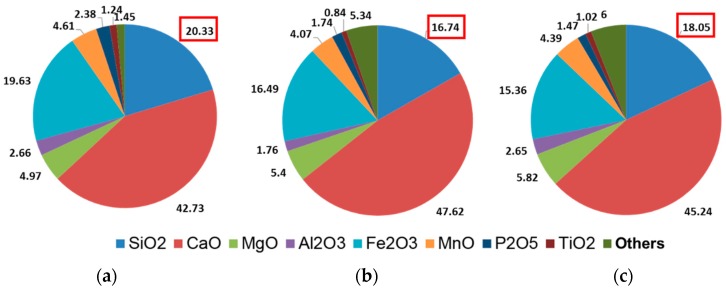
X-ray fluorescence spectrometer (XRF) results of different steel slags (**a**) LPSS; (**b**) RSS; (**c**) HBSS.

The XRF results of different steel slags are shown in Figure 5, it can be inferred that the chemical composition of three steel slags mainly contains CaO, SiO_2_ and Fe_3_O_4_, which accounts for more than 80% of the total composition. Compared with the main chemical composition of CaO obtained by the XRF test of traditional aggregate limestone, the steel slag contains a certain amount of silicate and iron-containing compounds due to the iron ore composition and the steel slag treatment process. Limestone is an excellent alkaline rock with good adhesion to asphalt binder. For steel slag, alkalinity which calculated by *M* = *w*(CaO)/[*w*(SiO_2_) + *w*(P_2_O_5_)], is used to evaluate the acidity and alkalinity of steel slag [32]. Wang Q divided BOF steel slags into three grades based on alkalinity: low alkalinity slag (*M* < 1.8), intermediate alkalinity slag (1.8 < *M* < 2.5) and high alkalinity slag (*M* > 2.5) [33]. Chen indicated that intermediate alkalinity slag and high alkalinity slag had better adhesion to asphalt binder [3]. Therefore, It can be seen from the XRF results, all three steel slags are medium-high alkalinity steel slags (*M* > 1.8) and has good adhesion to asphalt binder. In addition, *M* value of RSS is 26.84% higher than that of HBSS, and 9.72% higher than that of LPSS, which caused by the higher content of CaO and lower content of SiO_2_ in RSS.

### 3.2. Morphological Discrepancy

**Figure 6:**
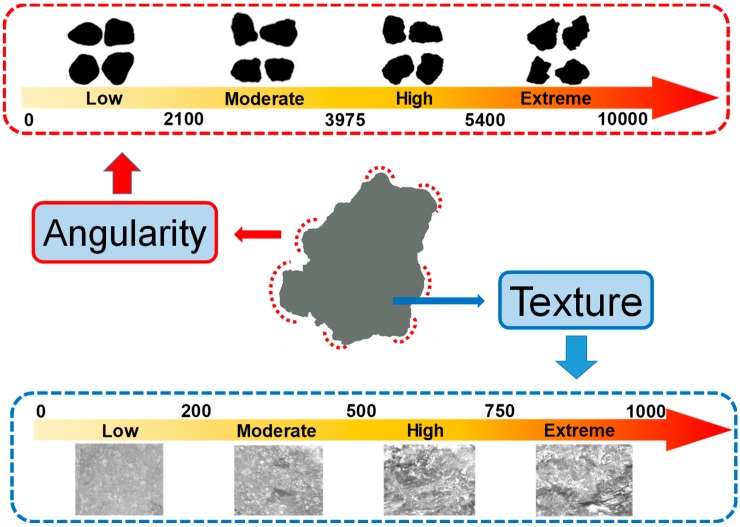
Schematic diagram of morphological properties.

Figure 6 shows the schematic diagram of morphological properties for coarse aggregates. Angularity is used to demonstrate variations at the corners, surface texture is used to describe the surface irregularity at a tiny scale to affect the overall shape. Because of different scales with respect to aggregate size, these two morphological properties can be distinguished and used to order them. Every property can be distinguished from other properties widely without necessarily affecting each other [28].

With regard to angularity, the angularity index is calculated by the radius method [34]. Through measuring the difference between particle radius in a certain direction and that of an equivalent ellipse, the angularity index can be calculated according to the following Equation (2) [28]:

(2) $$\mathrm{Angularity}\;\mathrm{Index}=\overset{355}{\underset{\theta =0}{\sum }}\frac{\left|R_{\theta }{-R}_{EE\theta }\right|}{R_{EE\theta }}$$

In the equation, *R_θ_* represents the radius of the particle at an angle of *θ*, and *R_EEθ_* represents the radius of the equivalent ellipse at an angle of *θ*. The equivalent ellipse has the same aspect ratio of the particle but has no angularity (smooth with no sharp corners). Normalization of the aspect ratio can minimize the effect of form on the angularity index [34]. The values of angularity index distribute from 0 to 10,000, and can also be divided into four levels: low level is from 0 to 2100 and particle is rounded, moderate level is from 2100 to 3975 and particle is sub-rounded, high level is from 3975 to 5400 and particle is sub-angular, extreme level is from 5400 to 10,000 and particle is angular. Additionally, the particle can be recognized to be more rounded if the angularity index is closer to 0.

**Figure 7:**
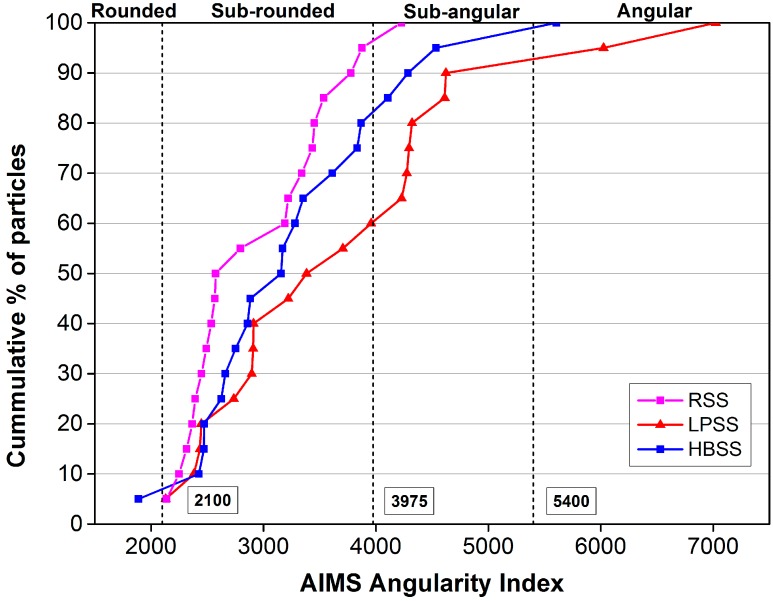
AIMS angularity index curve of tested steel slag particles.

Table 2 and Figure 7 summarize the angularity test results of different steel slags. It can be inferred that the angularity indexes of three steel slags are mainly distributed in the two levels of sub-rounded and sub-angular, and the distributions in sub-rounded account for the most. The average value and variance of the angularity of RSS are the smallest of three steel slags, while the average value and variance of LPSS are the largest. The results show that RSS owns the lowest angularity but the best control of variability. In contrast, LPSS owns the worst control of variability but the largest angularity.

Texture index is applied to characterize the texture distinction by wavelet analysis [35], the texture index at any given decomposition level is the arithmetic mean of the squared values of the detail coefficients of different level and can be calculated in the following Equation (3) [8]:

(3) $$\mathrm{Texture}\;\mathrm{Index}=\frac{1}{3N}\overset{3}{\underset{i=1}{\sum }}\overset{N}{\underset{j=1}{\sum }}{{(D}_{i,j}\left(x,y)\right)}^{2}$$

where *N* denotes the total number of coefficients in the detailed image of the aggregate; *i* takes a value of 1, 2, or 3, corresponding to three detailed images of the texture; J is the index of the wavelet coefficients, *D* means the transformed domain and (*x, y*) is the position of the coefficients in the texture scan area. The texture index can be divided into four levels: 0 to 200 accounts for the low grade, 200 to 500 accounts for the medium grade, 500 to 750 accounts for the high grade, and 750 to 1000 accounts for very high grade. Higher value of texture value represents the richer surface texture of aggregate.

**Figure 8:**
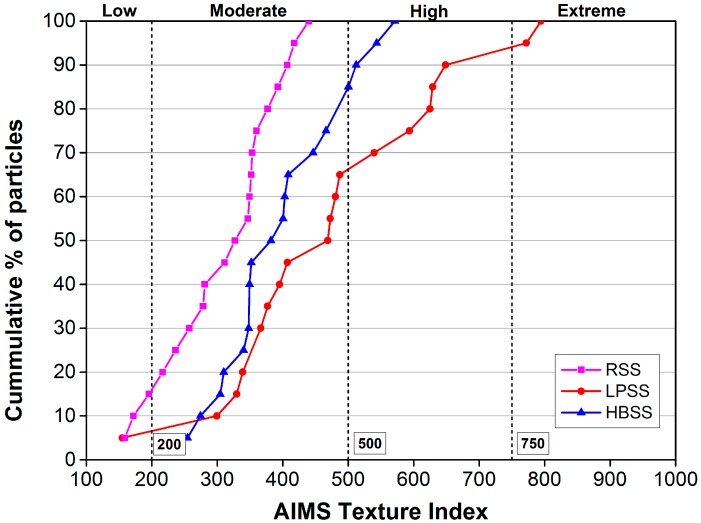
AIMS texture index curve of tested steel slag particles.

Table 3 and Figure 8 summarize the results of different steel slag texture indexes. Results show that the texture indexes of three steel slags are mainly distributed in the medium grade. Among the three steel slags, only the HBSS is not distributed in the low grade of texture index, only the LPSS is distributed in the high grade of texture index. As with the results of angular index, LPSS owns the highest texture index, and its average texture index is 53.24% and 19.06% higher than that of RSS and HBSS, respectively. However, the texture index value of LPSS is distributed in all four grades, its standard deviation of texture index is 94.83% and 78.58% higher than that of RSS and HBSS, respectively, showing poor texture variability.

Both the results of angular index and texture index show that LPSS has higher geometrical characteristics, this is mainly because that the cooling and treatment of RSS and HBSS experience both aging and stable processes in the initial formation of steel slag. Most free components have been digested into calcium carbonate, the composition and material properties of the steel slag have been relatively stable. However, cooling and treatment process of LPSS cannot guarantee that the aging of free components was finished completely during the slag splashing. Therefore, during the stacking period of LPSS, the free components continuously react with the moisture and carbon dioxide in the air, and the uneven distribution of aging products lead to the rugged surface texture of the aggregate, which increases the angular index and texture index of LPSS.

### 3.3. Road Performance

#### 3.3.1. High Temperature Stability 

The high temperature stability of asphalt pavement represents the ability of the asphalt mixture to resist permanent deformation after being subjected to vehicle load and repeated rolling under high temperature conditions. The rutting test is currently the mainstream method for evaluating the high temperature stability. If the high temperature stability of asphalt mixture does not meet the requirements of the standard design, the road surface will be damaged due to insufficient stability and low load loading rate at high temperature. The research shows that the dynamic stability of the rutting test has a good correlation with the rutting depth of the asphalt mixture [36]. By comparing the dynamic stability, the high temperature stability of the asphalt mixture can be evaluated.

**Figure 9:**
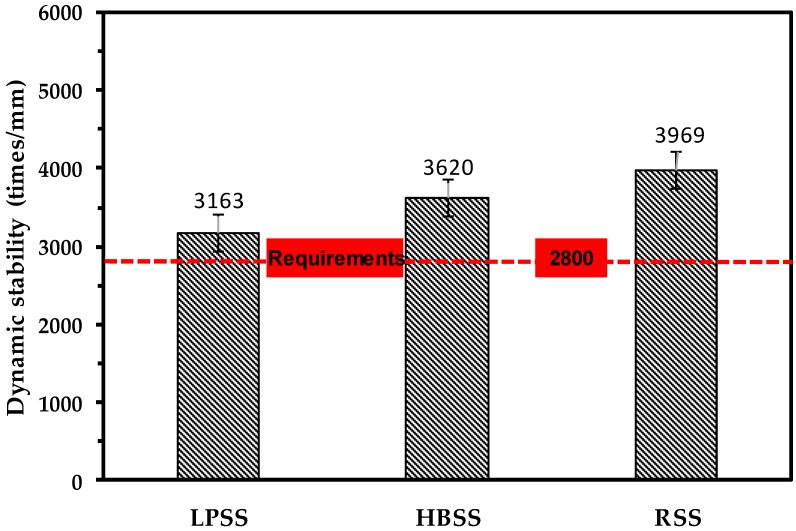
High-temperature stability test results of different steel slag asphalt mixtures.

Figure 9 shows the results of high temperature stability of different BOF steel slag asphalt mixture. It shows that the dynamic stability values of three steel slag asphalt mixtures are all higher than 2800 times/mm, which meets the requirements of the specification of China properly. Excellent material characteristics of BOF steel slag make its dynamic stability far greater than the specification requirements. Among them, the RSS asphalt mixture has the highest dynamic stability, which is 25.48% and 14.44% higher than that of LPSS asphalt mixture and HBSS asphalt mixture, respectively. The high temperature stability of LPSS is the worst, representing that although the LPSS has highest geometric parameters, it also has larger geometric variability and smaller crushing value, also the residual free material in LPSS affects the high temperature stability of asphalt mixture.

#### 3.3.2. Moisture Resistance Ability

**Figure 10:**
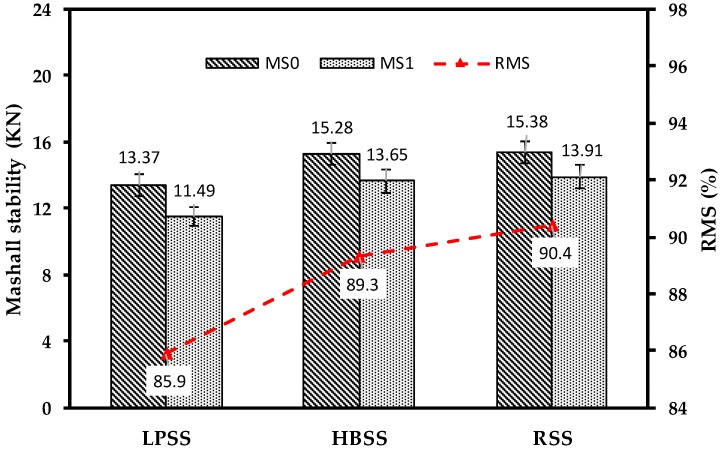
Water immersion Marshall test of Superpave-13 steel slag asphalt mixtures.

**Figure 11:**
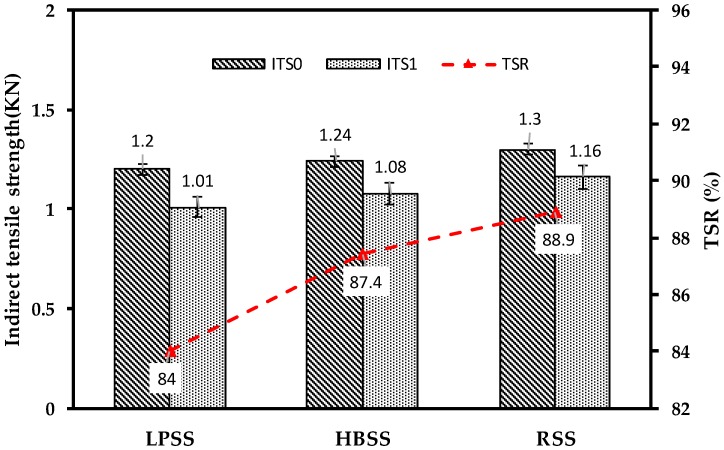
Freeze–thaw split test of Superpave-13 steel slag asphalt mixtures.

Moisture resistance is recognized as the ability to resist the peeling of bitumen, which adhering to the aggregate surface in asphalt mixture after being corroded by water. In this paper, according to ASTM D1075 and AASHTO T283, the moisture resistance abilities of steel slag asphalt mixtures were evaluated by the water immersion Marshall test and the freeze-thaw split test. The moisture resistance test results of Superpave-13 steel slag asphalt mixtures are shown in Figure 10 and Figure 11, it can be seen that the RMS values of three steel slag asphalt mixtures are all greater than 80%, and the TSR values of them were more than 75%, which are fully meeting the requirements of the specification. The RMS value of RSS asphalt mixture is 4.97% and 1.22% higher than that of LPSS asphalt mixture and HBSS asphalt mixture. In addition, the TSR value of RSS asphalt mixture is 5.51% and 1.68% higher than that of LPSS asphalt mixture and HBSS asphalt mixture. The moisture resistance test results of AC-13 steel slag asphalt mixtures are shown in Table 4. In consistent with the results of the Superpave-13 steel slag asphalt mixtures, the RMS values of three AC-13 steel slag asphalt mixture are all more than 80%, and the TSR values are greater than 75%. LPSS asphalt mixture still behaves the worst moisture resistance. Results show that three steel slag asphalt mixtures have good water stability, the main reason is that the steel slag shows coarse, porous surface texture that enhances the bonding performance between BOF steel slag and bitumen. In addition, the alkali metal cations such as Ca^2+^, Mg^2+^, Fe^2+^, Al^2+^ and Mn^2+^ contained in the steel slag can react with the bituminous acid, and the formed materials further increases the bonding property of the steel slag asphalt mixtures. In contrast to LPSS asphalt mixture and HBSS asphalt mixture, RSS asphalt mixture behaves the best moisture resistance, and the reason is that the more advanced treatment methods make the RSS more uniform and stable than the other two steel slags during aging process, and the produced silicate compounds (like CaCO_3_) improved the ability of asphalt mixture to resist water erosion.

#### 3.3.3. Low Temperature Cracking Resistance Ability

**Figure 12:**
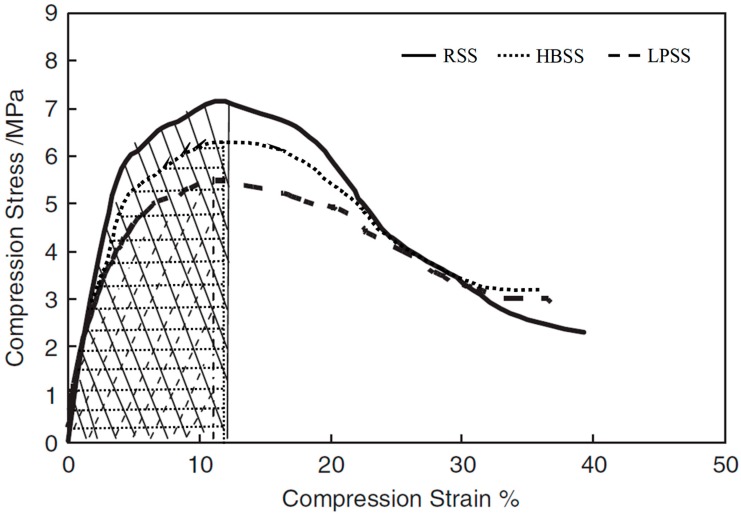
Compression stress–strain curves of AC-13 steel slag asphalt mixtures.

Low temperature crack resistance is the ability to characterize the asphalt mixture to resist temperature-shrinkage cracks at low temperatures. According to the measured relationship between compressive stress and strain of asphalt mixture shown in Equation (4) [14], it was found that the strain-stress relationship of asphalt mixture was quadratic parabola.

(4) $${\sigma =A\varepsilon }^{2}+B\varepsilon +C$$

where *σ* is the stress, MPa: *ε* is the strain; *A*, *B*, and *C* are constants, which are related to the type of material. So, the compressive strain energy density function can be transformed into the Equation (5) [17]:

(5) $$\frac{dW}{dV}=\int_{0}^{\varepsilon_{0}}\sigma_{ij}d\epsilon_{ij}=\frac{A}{3}\varepsilon_{0}{}^{3}{+B\varepsilon }_{0}{}^{2}{+C\varepsilon }_{0}$$

where *ε*_0_ is the strain at the peak point of compression stress–strain curve, and the stress–strain curves were obtained automatically by the data acquisition system. As shown in Figure 12, it can be clearly seen that the RSS asphalt mixture owns the largest *ε*_0_ while LPSS asphalt mixture has the lowest *ε*_0_. After substituting the *ε*_0_ values into the Equation (5), the critical value *dW/dV* is obtained in Table 5. Observations reveal that the critical value of *dW/dV* is 42.7 KJ/m^3^ for LPSS asphalt mixture, while RSS asphalt mixture owns the highest critical value of *dW/dV* and about 41.83% higher than that of LPSS asphalt mixture. Results confirm that LPSS asphalt mixture has the best ability to resist low temperature deformation.

### 3.4. Mechanism between Steel Slag Performance and Road Performance

**Figure 13:**
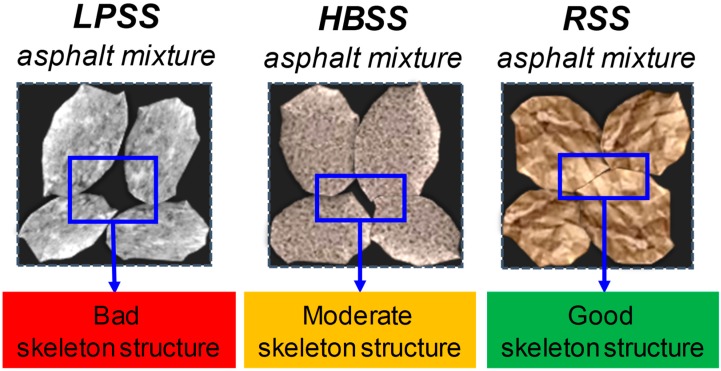
Structural sketch of different steel slag asphalt mixtures.

The road performance of steel slag asphalt mixture is closely related to the performance of steel slag. The reasons for the difference in road performance of asphalt mixtures prepared by different steel slag mainly include two aspects: the difference of the skeleton structure after the formation of steel slag asphalt mixture, and the difference of the adhesion performance between bitumen and steel slag. According to the results of the morphological discrepancy, Figure 13 shows the structural sketch of different steel slag asphalt mixtures. In morphological discrepancy section, RSS owns the best control of variability, in contrast, LPSS owns the worst control of variability. Thus, compared with HBSS and LPSS, RSS has a lower length-to-particle ratio and a smaller number of needle-like particles. At the same time, RSS has good angularity, while the shape variability is small, and the size between the particles is relatively balanced. Therefore, after the formation of asphalt mixtures, the RSS particles have more skeleton support points and are more likely to form a tight skeleton structure. The structure of RSS asphalt mixture is advantageous for improving the ability of the asphalt mixture to resist the deformation and enhancing the stability of structure.

**Figure 14:**
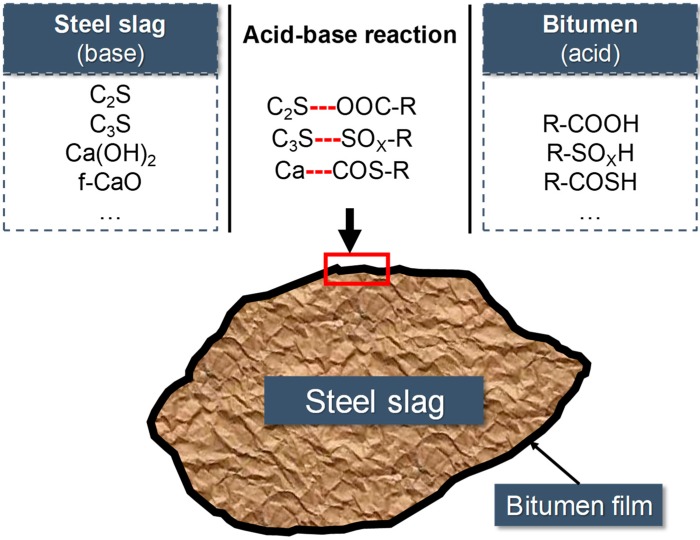
Schematic diagram of adhesion between steel slag and bitumen.

Figure 14 reveals the schematic diagram of adhesion between steel slag and bitumen. According to the XRF results, the BOF steel slag contains alkaline components like dicalcium silicate (C_2_S), tricalcium silicate (C_3_S), calcium hydroxide (Ca(OH)_2_), and free calcium oxide (f-CaO). The bitumen contains some acidic groups like R-COOH, R-SO_X_H, and R-COSH, which can react with the alkaline components in the steel slag to form an acid-base neutralization reaction, and the resulting product enhances the adhesion between the bitumen and steel slag. In addition, *M* value of RSS is 26.84% higher than that of HBSS, and 9.72% higher than that of LPSS, which caused by the higher content of CaO and lower content of SiO_2_ in RSS. This make the acid-base reaction of RSS asphalt mixture most intense, which contribute to the best road performance of it [5].

## 4. Conclusions

The primary purpose of this research was to examine the effects of different cooling and treatment processes on the morphological discrepancy of different BOF steel slag, including roller steel slag (RSS), hot braised steel slag (HBSS), and layer pouring steel slag (LPSS). Also, the road performances of corresponding asphalt mixtures were studied. On account of the results above, the following conclusions can be obtained.

RSS owns the lowest angularity but the best control of variability. In contrast, LPSS owns the worst control of variability but the highest angularity. Average texture index of LPSS is 53.24% and 19.06% higher than that of RSS and HBSS, respectively.

The RSS asphalt mixture has the highest dynamic stability, which is 25.48% and 14.44% higher than that of LPSS asphalt mixture and HBSS asphalt mixtures, respectively. The high temperature stability of LPSS asphalt mixture is the worst, representing that although the LPSS has higher geometric properties, it also has larger geometric variability and smaller crushing value, also the residual free material in LPSS affects the high temperature stability of asphalt mixture.

RSS asphalt mixture and LPSS asphalt mixture still behave the best and worst moisture resistance. Surface texture and alkali metal cations enhance the bonding performance between steel slag and bitumen. Observations reveal that the critical value of *dW/dV* is 42.7 KJ/m^3^ for LPSS asphalt mixture, while RSS asphalt mixture owns the highest critical value of *dW/dV* and about 41.83% higher than that of LPSS asphalt mixture.

The structure of RSS asphalt mixture is advantageous for improving the ability of the asphalt mixture to resist the deformation and enhancing the stability of structure. Higher content of CaO and lower content of SiO_2_ make the acid-base reaction of RSS asphalt mixture most intense, which contribute to the best road performance of it.

## Figures and Tables

**Table 1:** Basic properties of used aggregates. RSS: roller steel slag; LPSS: layer pouring steel slag; HBSS: hot braised steel slag.

Aggregate	Properties [21]					
	Size	Water Absorption (%)	Apparent Specific Gravity	Log Angeles Abrasion (%)	Crushing Value (%)	f-CaO Content (%)
RSS	9.5–16 mm	1.16	3.220	12.9	18.5	0.40
LPSS	9.5–16 mm	1.44	3.260	14.5	13.9	1.17
HBSS	9.5–16 mm	1.75	3.100	13.2	16.8	0.81
Basalt [22]	9.5–16 mm	0.40	2.774	16.8	20.0	N/A
Limestone [23]	9.5–16 mm	1.10	2.650	20.4	N/A	N/A
Granite [22]	9.5–16 mm	0.60	2.723	21.6	21.9	N/A
Limestone	4.75–9.5 mm	1.30	2.726	N/A	9.3	N/A
Limestone	2.36–4.75 mm	1.50	2.822	N/A	9.3	N/A
Requirements [24]	N/A	≤3.00	≥2.500	≤30.0	≤26.0	≤2.00

**Table 2:** Aggregate Imaging System (AIMS) angularity index of tested steel slag particles.

Angularity Index						
Sample	Average Value	Standard Deviation Value	Low (≤2100)	Moderate (2100–3975)	High (3975–5400)	Extreme (5400–10,000)
RSS	2946.5	609.6	0%	95%	5%	0%
HBSS	3292.0	862.2	5%	75%	15%	5%
LPSS	3725.8	1226.1	0	60%	30%	10%

**Table 3:** AIMS texture index values of tested steel slag particles.

Texture Index						
Sample	Average Value	Standard Deviation	Low (≤200)	Moderate (200–500)	High (500–750)	Extreme (750–1000)
RSS	311.4	81.3	15%	85%	0%	0%
HBSS	400.8	88.7	0%	75%	25%	0%
LPSS	477.2	158.4	5%	60%	25%	10%

**Table 4:** Moisture resistance results of AC-13 steel slag asphalt mixtures.

AC-13 Asphalt Mixture	RMS Test			ITS Test		
	MS_0_ (KN)	MS_1_ (KN)	RMS (%)	ITS_0_ (MPa)	ITS_1_ (MPa)	TSR (%)
RSS	16.92	15.59	92.1	1.65	1.49	90.3
HBSS	15.82	14.37	90.8	1.53	1.35	88.1
LPSS	14.10	12.44	88.2	1.49	1.28	85.6

**Table 5:** *ε*_0_ and critical value of *dW/dV* at peak poiont.

Mixture Type	Aggregate Type	*dW/dV*–Strain Function	*ε*_0_	Critical Value of *dW/dV* (KJ/m^3^)
AC-13 Asphalt mixture	LPSS	*dW/dV* = − 3 × 10 − 5*ε*^5^/5 + 0.0015*ε*^4^/4 − 0.0882*ε*^3^/3 + 1.128*ε*^2^/2 + 0.5047*ε*	12.0	42.7
	HBSS	*dW/dV =* − 3 × 10 − 5*ε*^5^/5 + 0.0019*ε*^4^/4 − 0.0904*ε*^3^/3 + 1.39*ε*^2^/2 + 0.6116*ε*	12.9	69.8
	RSS	*dW/dV =* − 3 × 10 − 5*ε*^5^/5 + 0.002*ε*^4^/4 − 0.0959*ε*^3^/3 + 1.44*ε*^2^/2 + 0.8109*ε*	13.3	73.4
